# Supplementary material for: Improving genetic risk modeling of dementia from real-world data in underrepresented populations
Source: Commun Biol. 2024 Aug 25;7:1049. doi: 10.1038/s42003-024-06742-0 (PMC11345412; doi:10.1038/s42003-024-06742-0)
Supplement: Supplementary file 2 — Supplemental Information [file 42003_2024_6742_MOESM2_ESM.pdf]

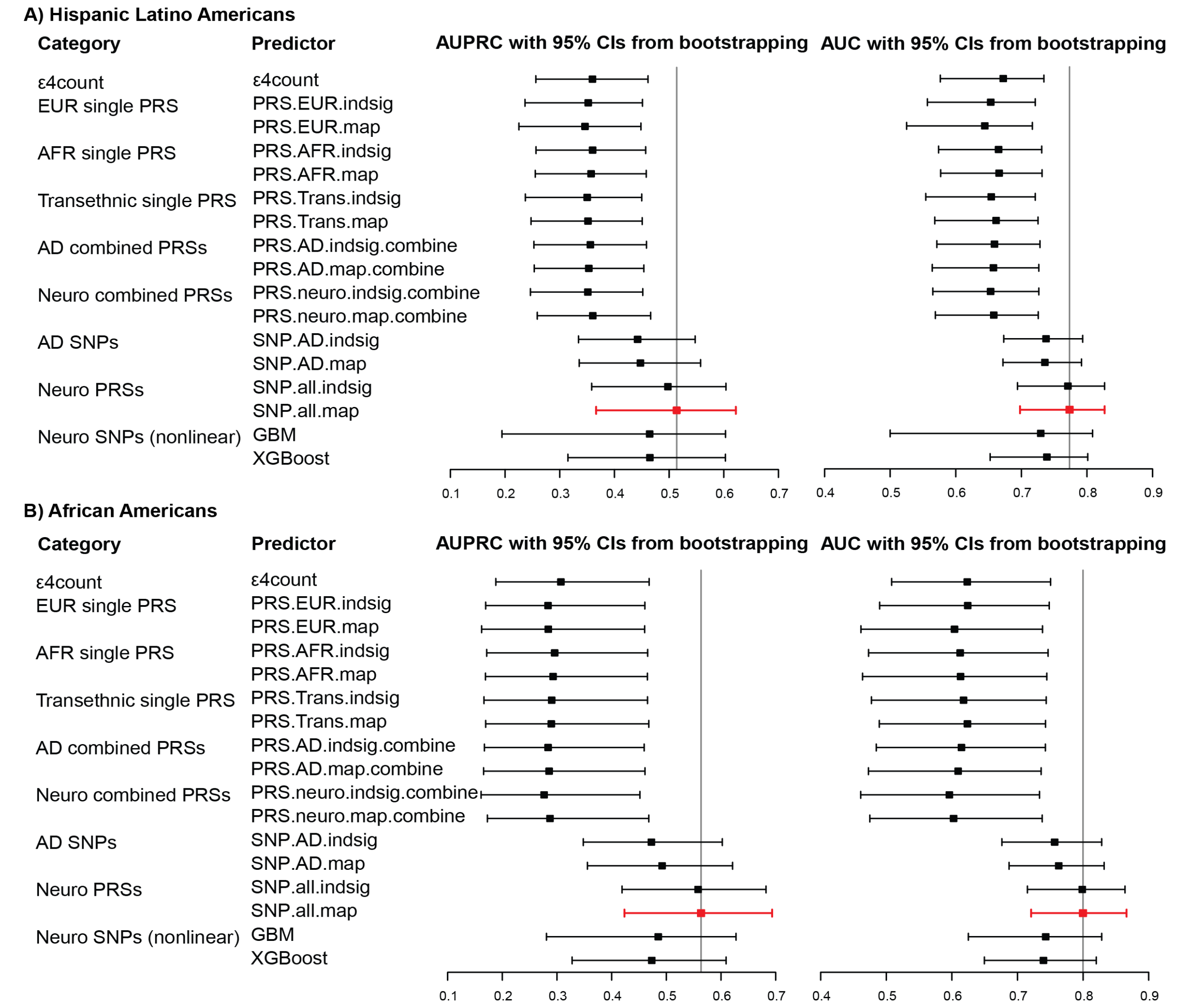

Supplementary Figure 1. Bootstrapping results on model performance of APOE-ε4 count, polygenic risk score, and machine learning SNP models in dementia genetic prediction, UCLA ATLAS sample, stratified by genetic inferred ancestry group. All models (if not other specified) have regressed out age, sex, and ancestry-specific principal components. Abbreviations: AD, Alzheimer's Disease; AUROC, Area Under the ROC Curve; AUPRC, Area Under the Precision-Recall Curve; EUR, European; PRS, Polygenic Risk Score; SNP, Single-Nucleotide Polymorphism.

Supplementary Table 1. Performance of models using lead SNPs (r^2 cut-off <0.1 for defining independent genome-wide-significant SNPs) in dementia genetic prediction, UCLA ATLAS sample, stratified by genetic

	Overall performace		Threshold maximize absolute MCC				
	AUPRC	AUROC	F1 score	Accuracy	Precision	Recall	Specificity
**Hispanic Latino American ancestry sample (N = 610)**							
**AD EUR PRS**	0.284	0.594	0.311	**0.761**	**0.384**	0.262	**0.890**
**AD AFR PRS**	0.314	0.651	0.418	0.716	0.363	0.492	0.775
**AD multi-ancestry PRS**	0.304	0.623	0.375	0.693	0.324	0.444	0.758
**PRSs using AD GWASs only**[b]	0.308	0.640	0.419	0.705	0.353	0.516	0.754
**PRSs using AD + Neuro GWASs**[c]	0.298	0.624	0.391	0.703	0.339	0.460	0.767
**SNPs from AD GWASs only**	0.343	0.684	0.431	0.602	0.306	0.730	0.568
**SNPs from AD + Neuro GWASs**	**0.357**	**0.695**	**0.435**	0.549	0.294	**0.841**	0.473
**African American ancestry sample (N = 440)**							
	**Overall performace**		**Threshold maximize absolute MCC**				
	**AUPRC**	**AUROC**	**F1 score**	**Accuracy**	**Precision**	**Recall**	**Specificity**
**AD EUR PRS**	0.206	0.549	0.322	0.627	0.247	0.464	0.666
**AD AFR PRS**	0.254	0.590	0.342	0.711	0.303	0.393	0.787
**AD multi-ancestry PRS**	0.264	0.604	0.368	0.516	0.245	0.738	0.463
**PRSs using AD GWASs only**[b]	0.283	0.591	0.250	**0.809**	**0.500**	0.167	**0.961**
**PRSs using AD + Neuro GWASs**[c]	0.240	0.572	0.343	0.295	0.209	**0.964**	0.138
**SNPs from AD GWASs only**	0.281	0.647	0.409	0.691	0.322	0.560	0.722
**SNPs from AD + Neuro GWASs**	**0.327**	**0.683**	**0.432**	0.755	0.387	0.488	0.817

Abbreviations: AD, Alzheimer's Disease; AFR, African American; AUROC, Area Under the ROC Curve; AUPRC, Area Under the Precision-Recall Curve; EUR, European; GWAS, Genome-Wide Association Study; MCC, Matthews Correlation Coefficient; PRS, Polygenic Risk Score; SNP, Single-Nucleotide Polymorphism.

Notes:

[a] All models (if not other specified) adjusted for age, sex, and first four ancestry-specific principal components.

[b] All AD PRSs built with EUR, AFR, and multi-ancestry GWASs using P-significant/gene-annotated SNPs were included in the model at the same time.

[c] All AD PRSs built with EUR, AFR, and multi-ancestry GWASs, and neurodegenerative disease PRS (Parkinson's disease, progressive supranuclear palsy, Lewy body dementia, and stroke) using P-significant/gene-annotated SNPs were included in the model at the same time.

Supplementary Table 2. Full featured risk SNPs from the best-performing Elastic Net SNP model, UCLA ATLAS sample, stratified by genetic ancestry

No.	rsID	CHR	POS	Variable Importanc	Nearest Gene	Distance	Function	CADD	Position Map	eQTL Map	CI Map	AD_EUR	AD_AFR	AD_trans	LBD	PD	PSP	STROKE
**Hispanic Latino American ancestry (HLA)**																		
1	rs429358	19	44908684	0.089 (0.02, 0.11)	*APOE*	0	exonic	12.64	1	0	1		x					
2	rs2075650	19	44892362	0.089 (0.02, 0.11)	*TOMM40*	0	intronic	7.974	1	0	1		x	x	x			
3	rs483082	19	44912921	0.07 (0.017, 0.086)	*APOC1*	1325	intergenic	11.01	1	0	1		x	x				
4	rs157581	19	44892457	0.065 (0.014, 0.079)	*TOMM40*	0	exonic	21.1	1	0	1		x		x			
5	rs412776	19	44876259	0.057 (0.016, 0.066)	*NECTIN2*	0	intronic	12.66	1	0	0	x		x				
6	rs62120578	19	44713297	0.053 (0.022, 0.061)	*CTB-171A8.1*	0	ncRNA_intronic	12.82	1	0	0	x						
7	rs4803765	19	44855191	0.048 (0.015, 0.056)	*NECTIN2*	0	intronic	1.401	1	0	0	x						
8	rs80100206	4	705856	0.042 (0.02, 0.046)	*PCGF3*	0	UTR5	19.73	1	1	0					x		
9	rs6857	19	44888997	0.038 (0.011, 0.042)	*NECTIN2*	0	ncRNA_intronic	5.597	1	0	1		x					
10	rs2276412	11	121590137	0.037 (0.018, 0.039)	*SORL1*	0	exonic	14.94	1	0	0	x						
11	rs2220427	4	110793733	0.033 (0.019, 0.035)	*RP11-777N19.1*	0:00	upstream	20.4	1	0	0						x	
12	rs117783785	19	45195929	0.03 (0.016, 0.032)	*BLOC1S3*	0	intronic	6.441	1	0	0	x						
13	rs7026	19	44821259	0.027 (0.013, 0.029)	*BCAM*	0	UTR3	11.62	1	0	0	x		x	x			
14	rs80335384	17	45321252	0.027 (0.017, 0.029)	*MAP3K14*	4203	intergenic	9.95	1	1	1							x
15	rs2627641	19	45205500	0.026 (0.014, 0.028)	*BLOC1S3*	0:00:00	intronic	11.65	1	0	0	x						
16	rs435380	19	44903861	0.025 (0.014, 0.028)	*TOMM40*	171	downstream	9.048	1	1	0		x	x				
17	rs13067212	3	39404095	0.025 (0.018, 0.028)	*RPSA*	2593	intergenic	13.8	1	1	0							x
18	rs1551890	19	44829875	0.025 (0.01, 0.028)	*BCAM*	8458	intergenic	8.368	1	0	0	x		x				
19	rs10422350	19	44725238	0.023 (0.016, 0.026)	*snoZ6*	745	downstream	13.12	1	0	0	x		x				
20	rs7613	17	45394115	0.02 (0.016, 0.024)	*ARHGAP27*	0	UTR3	15.45	1	1	1							x
21	rs56870644	19	11065610	0.019 (0.016, 0.023)	*SMARCA4*	214	downstream	11.62	1	0	0						x	
22	rs76692773	19	44890954	0.019 (0.013, 0.023)	*TOMM40*	0:00	intronic	7.614	1	0	1	x		x	x			
23	rs35929607	2	168179226	0.012 (0.007, 0.019)	*STK39*	0	intronic	16.92	1	0	1					x		
24	rs17030651	1	112696760	0.012 (0.006, 0.018)	*MOV10*	0	exonic	19.29	1	1	0						x	
25	rs11223641	11	133950127	0.011 (0.004, 0.018)	*IGSF9B*	0	intronic	10.74	1	0	1					x		
26	rs10401176	19	44750234	0.009 (0.002, 0.018)	*BCL3*	0	intronic	13.68	1	0	0	x		x				
27	rs2619363	4	89837896	0.006 (0.003, 0.014)	*SNCA*	0	ncRNA_intronic	21.6	1	1	0				x	x		
28	rs28399637	19	44820881	0.004 (0, 0.012)	*BCAM*	0	intronic	0.114	1	0	0	x		x	x			
**African American ancestry (AA)**																		
1	rs2627641	19	45205500	0.096 (0.077, 0.099)	*BLOC1S3*	0:00	intronic	11.65	1	0	0	x						
2	rs8073976	17	44955857	0.079 (0.065, 0.082)	*C1QL1*	3835	intergenic	16.04	1	1	1						x	
3	rs77283277	7	143386852	0.076 (0.063, 0.079)	*ZYX*	0	intronic	5.616	1	1	1	x						
4	rs429358	19	44908684	0.071 (0.059, 0.074)	*APOE*	0	exonic	12.64	1	0	1		x					
5	rs2075650	19	44892362	0.068 (0.056, 0.071)	*TOMM40*	0	intronic	7.974	1	0	1		x	x	x			
6	rs73936967	19	44890485	0.064 (0.053, 0.066)	*TOMM40*	0	ncRNA_intronic	22	1	0	1		x					
7	rs13032148	2	127107524	0.063 (0.053, 0.065)	*BIN1*	168	upstream	7.853	1	0	0	x		x				
8	rs71352239	19	44926286	0.056 (0.047, 0.057)	*APOC1P1*	517	upstream	5.237	1	1	1	x		x	x			
9	rs435380	19	44903861	0.041 (0.037, 0.042)	*TOMM40*	171	downstream	9.048	1	1	0		x	x				
10	rs11223641	11	133950127	0.041 (0.037, 0.041)	*IGSF9B*	0	intronic	10.74	1	0	1					x		
11	rs2238682	19	44955336	0.036 (0.033, 0.036)	*CLPTM1*	0	UTR5	17.4	1	1	1	x		x				
12	rs73572039	19	44839373	0.034 (0.032, 0.034)	*NECTIN2*	6801	intergenic	4.628	1	0	0			x				
13	rs3800298	6	43284291	0.033 (0.032, 0.034)	*TTBK1*	0	exonic	15.75	1	0	0							x
14	rs28399635	19	44819986	0.028 (0.027, 0.028)	*BCAM*	0	UTR3	4.212	1	0	0	x		x				
15	rs4803791	19	45020325	0.023 (0.022, 0.024)	*RELB*	0	intronic	7.817	1	1	1	x		x				
16	rs41289510	19	44844379	0.022 (0.021, 0.023)	*NECTIN2*	1795	intergenic	3.223	1	0	0	x		x				
17	rs2889414	19	44794671	0.02 (0.018, 0.022)	*CBLC*	0:00	intronic	3.046	1	1	0	x		x				
18	rs75178253	19	44957750	0.02 (0.018, 0.022)	*CLPTM1*	0	intronic	0.32	1	0	1	x		x				
19	rs3810143	19	44846145	0.018 (0.016, 0.02)	*NECTIN2*	29	upstream	8.297	1	0	0	x		x				

20	rs4263041	19	44935386	0.014 (0.013, 0.016)	*APOC1P1*	3999	intergenic	2.278	1	0	1	x					
21	rs76692773	19	44890954	0.012 (0.012, 0.015)	*TOMM40*	0	intronic	7.614	1	0	1	x		x	x		
22	rs166907	19	44883598	0.01 (0.009, 0.013)	*NECTIN2*	0	ncRNA_intronic	3.815	1	0	0		x	x			
23	rs62330386	4	154525388	0.009 (0.009, 0.014)	*RP11-158C21.2*	580	downstream	5.44	1	0	0						x
24	rs12981508	19	44855243	0.009 (0.008, 0.013)	*NECTIN2*	0	intronic	1.322	1	0	0	x		x			
25	rs13067212	3	39404095	0.008 (0.007, 0.014)	*RPSA*	2593	intergenic	13.8	1	1	0					x	
26	rs71352248	19	44999245	0.008 (0.007, 0.013)	*RELB*	2184	intergenic	15.03	1	0	0	x					
27	rs76738189	19	45216235	0.007 (0.007, 0.013)	*EXOC3L2*	0	intronic	3.762	1	0	0	x					
28	rs157581	19	44892457	0.007 (0.006, 0.011)	*TOMM40*	0	exonic	21.1	1	0	1		x		x		
29	rs3852860	19	44879709	0.007 (0.006, 0.012)	*NECTIN2*	0	intronic	4.255	1	0	0	x		x			
30	rs73936968	19	44892559	0.004 (0.003, 0.011)	*TOMM40*	0	intronic	8.817	1	0	1	x					
31	rs2304933	11	60335034	0.002 (0.001, 0.009)	*MS4A6E*	0	exonic	17.97	1	0	0	x					

Abbreviations: AD, Alzheimer's Disease; AFR, African American; CI, confidence interval; EUR, European; LBD, Lewy body dementia; PD, Parkinson's disease; PRS, Polygenic Risk Score; PSP, progressive supranuclear palsy; SNP, Single-Nucleotide Polymorphism. Note: SNPs marked in red are overlapped SNPs identified by both samples.

Supplementary Table 3. Mapped genes of selected risk SNPs from the best-performing Elastic Net SNP model, UCLA ATLAS sample, by genetic inferred ancestry

No	Gene	Symbol	CHR	Start	End	Type	posMap	eqtlMap (Direction)	ciMap
**Hispanic Latino Americans (HLA)**									
1	ENSG00000134245	*WNT2B*	1	113009163	113072787	protein_coding	No	Yes (+)	No
2	ENSG00000007341	*ST7L*	1	113066140	113163447	protein_coding	No	Yes (+)	No
3	ENSG00000155363	*MOV10*	1	113215763	113243368	protein_coding	Yes	No	No
4	ENSG00000198648	*STK39*	2	168810530	169104651	protein_coding	Yes	No	Yes
5	ENSG00000144659	*SLC25A38*	3	39424839	39438842	protein_coding	No	Yes (+)	No
6	ENSG00000168028	*RPSA*	3	39448180	39454033	protein_coding	Yes	Yes (+)	No
7	ENSG00000185619	*PCGF3*	4	699537	764428	protein_coding	Yes	Yes (-)	No
8	ENSG00000247775	*RP11-67M1.1*	4	90757559	90763129	antisense	No	Yes (-)	No
9	ENSG00000247775	*RP11-67M1.1*	4	90757559	90763129	antisense	No	Yes (+)	No
10	ENSG00000249519	*RP11-777N19.1*	4	111715559	111718500	lincRNA	Yes	No	No
11	ENSG00000137642	*SORL1*	11	121322912	121504402	protein_coding	Yes	No	No
12	ENSG00000080854	*IGSF9B*	11	133778459	133826880	protein_coding	Yes	No	No
13	ENSG00000255406	*RP11-713P17.5*	11	133896882	133898013	lincRNA	No	No	Yes
14	ENSG00000166086	*JAM3*	11	133938820	134021896	protein_coding	No	No	Yes
15	ENSG00000254481	*PTP4A2P2*	11	133993723	133994224	pseudogene	No	No	Yes
16	ENSG00000180329	*CCDC43*	17	42750437	42767147	protein_coding	No	No	Yes
17	ENSG00000184922	*FMNL1*	17	43298811	43324687	protein_coding	No	Yes (+)	No
18	ENSG00000184361	*SPATA32*	17	43331760	43339479	protein_coding	No	No	Yes
19	ENSG00000006062	*MAP3K14*	17	43340488	43394414	processed_transcript	Yes	No	Yes
20	ENSG00000267446	*CTB-39G8.2*	17	43448768	43449423	lincRNA	No	No	Yes
21	ENSG00000159314	*ARHGAP27*	17	43471275	43511787	protein_coding	Yes	Yes (+)	No
22	ENSG00000159314	*ARHGAP27*	17	43471275	43511787	protein_coding	Yes	Yes (-)	No
23	ENSG00000267344	*CTB-39G8.3*	17	43474298	43474843	antisense	No	No	Yes
24	ENSG00000225190	*PLEKHM1*	17	43513266	43568115	protein_coding	No	Yes (+)	No
25	ENSG00000214425	*LRRC37A4P*	17	43578685	43627701	pseudogene	No	Yes (+)	No
26	ENSG00000264070	*DND1P1*	17	43663237	43664295	pseudogene	No	Yes (-)	No
27	ENSG00000263503	*RP11-707O23.5*	17	43678235	43679706	pseudogene	No	Yes (-)	No
28	ENSG00000204650	*CRHR1-IT1*	17	43697694	43725582	pseudogene	No	Yes (-)	No
29	ENSG00000185294	*SPPL2C*	17	43922256	43924438	protein_coding	No	Yes (-)	No
30	ENSG00000214401	*KANSL1-AS1*	17	44270942	44274089	antisense	No	Yes (-)	No
31	ENSG00000262500	*RP11-259G18.2*	17	44320972	44322410	pseudogene	No	Yes (-)	No
32	ENSG00000262539	*RP11-259G18.3*	17	44336917	44337972	pseudogene	No	Yes (-)	No
33	ENSG00000261575	*RP11-259G18.1*	17	44344403	44346060	pseudogene	No	Yes (-)	Yes
34	ENSG00000127616	*SMARCA4*	19	11071598	11176071	protein_coding	Yes	No	No
35	ENSG00000266903	*CTB-171A8.1*	19	45135500	45222031	antisense	Yes	No	No
36	ENSG00000252200	*snoZ6*	19	45229248	45229322	snoRNA	Yes	No	No
37	ENSG00000069399	*BCL3*	19	45250962	45263301	protein_coding	Yes	No	No
38	ENSG00000187244	*BCAM*	19	45312328	45324673	protein_coding	Yes	No	No
39	ENSG00000130202	*PVRL2*	19	45349432	45392485	protein_coding	Yes	No	No
40	ENSG00000130204	*TOMM40*	19	45393826	45406946	protein_coding	Yes	No	No
41	ENSG00000130203	*APOE*	19	45409011	45412650	protein_coding	Yes	Yes (-)	No
42	ENSG00000130208	*APOC1*	19	45417504	45422606	protein_coding	Yes	No	No
43	ENSG00000214855	*APOC1P1*	19	45430061	45434643	pseudogene	No	No	Yes
44	ENSG00000104859	*CLASRP*	19	45542298	45574214	protein_coding	No	No	Yes
**African Americans (AA)**									
**No**	**Gene**	**Symbol**	**CHR**	**Start**	**End**	**Type**	**posMap**	**eqtlMap (Direction)**	**ciMap**
1	ENSG00000136717	*BIN1*	2	127805603	127864931	protein_coding	Yes	No	No
2	ENSG00000144659	*SLC25A38*	3	39424839	39438842	protein_coding	No	Yes (+)	No
3	ENSG00000168028	*RPSA*	3	39448180	39454033	protein_coding	Yes	Yes (+)	No
4	ENSG00000250609	*RP11-158C21.2*	4	155444639	155445959	pseudogene	Yes	No	No
5	ENSG00000146216	*TTBK1*	6	43211418	43255997	protein_coding	Yes	No	No
6	ENSG00000159840	*ZYX*	7	143078173	143088204	protein_coding	Yes	No	No
7	ENSG00000050327	*ARHGEF5*	7	144052381	144077725	protein_coding	No	Yes (+)	No
8	ENSG00000166926	*MS4A6E*	11	60102304	60164069	protein_coding	Yes	No	No
9	ENSG00000080854	*IGSF9B*	11	133778459	133826880	protein_coding	Yes	No	No
10	ENSG00000255406	*RP11-713P17.5*	11	133896882	133898013	lincRNA	No	No	Yes
11	ENSG00000166086	*JAM3*	11	133938820	134021896	protein_coding	No	No	Yes
12	ENSG00000254481	*PTP4A2P2*	11	133993723	133994224	pseudogene	No	No	Yes
13	ENSG00000180329	*CCDC43*	17	42750437	42767147	protein_coding	No	No	Yes
14	ENSG00000161692	*DBF4B*	17	42785976	42829632	protein_coding	No	Yes (-)	No
15	ENSG00000073670	*ADAM11*	17	42836399	42859214	protein_coding	No	No	Yes
16	ENSG00000131095	*GFAP*	17	42982376	42994305	protein_coding	No	No	Yes
17	ENSG00000131094	*C1QL1*	17	43037061	43045439	protein_coding	Yes	Yes (+)	No
18	ENSG00000267788	*CTD-2534I21.9*	17	43059882	43060140	lincRNA	No	No	Yes
19	ENSG00000266903	*CTB-171A8.1*	19	45135500	45222031	antisense	No	Yes (+)	No
20	ENSG00000142273	*CBLC*	19	45281126	45303891	protein_coding	Yes	No	No
21	ENSG00000187244	*BCAM*	19	45312328	45324673	protein_coding	Yes	No	No
22	ENSG00000130202	*PVRL2*	19	45349432	45392485	protein_coding	Yes	No	No
23	ENSG00000267282	*CTB-129P6.4*	19	45385284	45394133	antisense	Yes	No	No
24	ENSG00000130204	*TOMM40*	19	45393826	45406946	protein_coding	Yes	No	No
25	ENSG00000130203	*APOE*	19	45409011	45412650	protein_coding	Yes	Yes (-)	No
26	ENSG00000130208	*APOC1*	19	45417504	45422606	protein_coding	No	No	Yes
27	ENSG00000214855	*APOC1P1*	19	45430061	45434643	pseudogene	Yes	Yes (+)	Yes
28	ENSG00000234906	*APOC2*	19	45449243	45452822	protein_coding	No	Yes (+)	No
29	ENSG00000104853	*CLPTM1*	19	45457842	45496599	protein_coding	Yes	No	No
30	ENSG00000104856	*RELB*	19	45504688	45541452	protein_coding	Yes	No	No
31	ENSG00000104859	*CLASRP*	19	45542298	45574214	protein_coding	No	No	Yes
32	ENSG00000267045	*AC006126.4*	19	45741890	45748628	lincRNA	No	No	Yes

Supplementary Table 4A. Descriptive statistics of demographic and electronic health record features by case/control groups, UCLA ATLAS sample (East Asian American ancestry group, N = 673)

	**Cases**	**Controls**	**P value**
N	75	598	-
Age	80.1 (73.2, 83.5)	76.3 (72.9, 80.7)	0.04*
Sex (Female)	48 (64%)	325 (54%)	0.11
Span of records (in yrs)	5.3 (2.7, 7.8)	9.8 (8.0, 12.0)	<0.001*
Encounters per year	17 (9, 28)	12 (7, 18)	0.002*
Number of encounters	73 (23, 137)	121 (68, 185)	<0.001*
Number of unique diagnosis	64 (35, 92)	60 (38, 90)	0.80

Abbreviations: EHR, electronic health record.

Notes: Continuous variables were reported as median (IQR), and categorical variables were reported as n (%). P-values were calcualted based on Wilcoxon rank sum test or Pearson's Chi-squared test as appropriate. * Statistical siginificant at level 0.05.

Supplementary Table 4B. Model performance of *APOE-ε4* count, polygenic risk score, and Elastic Net SNP models in dementia genetic prediction, UCLA ATLAS sample (East Asian American ancestry group, N = 673)[a]

		AUPRC	AUROC	F1 score	Accuracy	Precision	Recall	Specificity
**APOE**	ε4 count	0.238	0.712	0.347	0.782	0.260	0.520	0.814
			**AD-PRS models**					
**AD EUR PRS**	P-significant	0.222	0.681	0.252	0.877	**0.389**	0.187	**0.963**
	Gene-annotated	0.215	0.676	0.262	0.866	0.340	0.213	0.948
**AD AFR PRS**	P-significant	0.234	0.706	0.336	0.765	0.245	0.533	0.794
	Gene-annotated	0.230	0.700	0.331	0.765	0.242	0.520	0.796
**AD multi-ancestry PRS**	P-significant	0.236	0.702	0.316	0.730	0.220	0.560	0.751
	Gene-annotated	0.232	0.696	0.318	0.796	0.254	0.427	0.843
			**Multi-PRS models**					
**PRSs using AD GWASs only**[b]	P-significant	0.234	0.699	0.327	0.731	0.227	0.587	0.749
	Gene-annotated	0.229	0.700	0.338	0.779	0.253	0.507	0.813
**PRSs using AD + Neuro GWASs**[c]	P-significant	0.234	0.704	0.338	0.796	0.265	0.467	0.838
	Gene-annotated	0.224	0.706	0.346	0.770	0.253	0.547	0.798
			**Elastic Net SNPs models**					
**SNPs from AD GWASs only**	P-significant	0.234	0.706	0.350	0.774	0.258	0.547	0.803
	Gene-annotated	0.256	0.743	0.338	0.685	0.220	0.720	0.681
**SNPs from AD + Neuro GWASs**	P-significant	0.271	0.748	0.366	0.835	0.320	0.427	0.886
	Gene-annotated	**0.338**	**0.793**	**0.443**	**0.840**	0.361	0.573	0.873
			**Non-linear SNPs models**					
**SNPs from AD + Neuro GWASs**	GBM	0.113	0.511	0.219	0.416	0.129	**0.733**	0.376
**Gene-annotated SNPs**	XGBoost	0.206	0.673	0.300	0.660	0.194	0.653	0.661

Abbreviations: AD, Alzheimer's Disease; APOE, apolipoprotein E; AUROC, Area Under the ROC Curve; AUPRC, Area Under the Precision-Recall Curve; EUR, European; GBM, Gradient Boosting Machine; GWAS, Genome-Wide Association Study; PRS, Polygenic Risk Score; SNP, Single-Nucleotide Polymorphism.

Notes:

[a] All models (if not other specified) have regressed out age, sex, and ancestry-specific principal components. Thresholds were determined by maximizing absolute Matthews correlation coefficient.

[b] All AD PRSs built with EUR, AFR, and multi-ancestry GWASs using P-significant/gene-annotated SNPs were included in the model at the same time.

[c] All AD PRSs built with EUR, AFR, and multi-ancestry GWASs, and neurodegenerative disease PRS (Parkinson's disease, progressive supranuclear palsy, Lewy body dementia, and stroke) using P-significant/gene-annotated SNPs were included in the model at the same time.

Supplementary Table 4C. Selected risk SNPs from the best-performing Elastic Net SNP model, UCLA ATLAS sample (East Asian American ancestry group, N = 673)

rsID	CHR	POS	Variable Importance (percentage, 95% CI)	Nearest Gene	AD EUR	AD AFR	AD multi	LBD	PD	PSP	Stroke
rs429358	19	44908684	0.205 (0.151, 0.214)	*APOE*		x					
rs66626994	19	44924977	0.133 (0.104, 0.137)	*APOC1P1*			x	x			
rs6857	19	44888997	0.118 (0.095, 0.122)	*NECTIN2*		x					
rs2075650	19	44892362	0.101 (0.084, 0.104)	*TOMM40*		x	x	x			
rs483082	19	44912921	0.092 (0.078, 0.094)	*APOC1*		x	x				
rs35106910	19	44781009	0.079 (0.07, 0.081)	*CBLC*	x						
rs1105568	17	45716061	0.05 (0.049, 0.051)	*CRHR1*						x	
rs76738189	19	45216235	0.049 (0.049, 0.051)	*EXOC3L2*	x						
rs34096562	8	16843772	0.047 (0.046, 0.049)	*RP11-13N12.1*					x		
rs7932740	11	60208638	0.029 (0.028, 0.038)	*MS4A4E*	x		x				
rs11724804	4	971991	0.021 (0.019, 0.032)	*DGKQ*				x	x		
rs13067212	3	39404095	0.019 (0.017, 0.031)	*RPSA*						x	
rs7613	17	45394115	0.018 (0.016, 0.03)	*ARHGAP27*						x	
chr1:207368589:D	1	207195244	0.015 (0.012, 0.028)	*RP11-164O23.7*	x						
rs59193782	17	45357199	0.01 (0.008, 0.025)	*CTB-39G8.2*						x	
rs10769263	11	47395632	0.007 (0.004, 0.023)	*RP11-750H9.5*	x						

Abbreviations: AD, Alzheimer's Disease; AFR, African American; CI, confidence interval; EUR, European; LBD, Lewy body dementia; PD, Parkinson's disease; PRS, Polygenic Risk Score; PSP, progressive supranuclear palsy;SNP, Single-Nucleotide Polymorphism.

Supplementary Table 4D. Mapped genes of selected risk SNPs from the best-performing Elastic Net SNP model, UCLA ATLAS sample (East Asian American ancestry group, N = 673)

No	Gene	Symbol	CHR	Start	End	Type	posMap	eqtlMap (Direction)	ciMap
1	ENSG00000203710	*CR1*	1	207669492	207813992	protein_coding	No	Yes (+)	No
2	ENSG00000127419	*TMEM175*	4	926175	952444	protein_coding	No	Yes (-)	No
3	ENSG00000145214	*DGKQ*	4	952675	980683	protein_coding	Yes	Yes (-)	No
4	ENSG00000145217	*SLC26A1*	4	972861	987228	protein_coding	No	Yes (+)	No
5	ENSG00000253496	*RP11-13N12.1*	8	16534414	16772553	lincRNA	Yes	No	No
6	ENSG00000175220	*ARHGAP1*	11	46698630	46722165	protein_coding	No	Yes (+)	No
7	ENSG00000255197	*RP11-750H9.5*	11	47404699	47430741	antisense	Yes	Yes (-)	No
8	ENSG00000165915	*SLC39A13*	11	47428683	47438047	protein_coding	No	Yes (-)	No
9	ENSG00000213619	*NDUFS3*	11	47586888	47606114	protein_coding	No	Yes (-)	No
10	ENSG00000196666	*FAM180B*	11	47608198	47610746	protein_coding	No	Yes (-)	No
11	ENSG00000184922	*FMNL1*	17	43298811	43324687	protein_coding	No	Yes (+)	No
12	ENSG00000233175	*CTD-2020K17.3*	17	43315395	43319101	antisense	No	Yes (+)	No
13	ENSG00000006062	*MAP3K14*	17	43340488	43394414	processed_transcript	No	No	Yes
14	ENSG00000199953	*RNA5SP443*	17	43404732	43404863	rRNA	Yes	No	No
15	ENSG00000267446	*CTB-39G8.2*	17	43448768	43449423	lincRNA	Yes	No	No
16	ENSG00000159314	*ARHGAP27*	17	43471275	43511787	protein_coding	Yes	Yes (-)	No
17	ENSG00000214425	*LRRC37A4P*	17	43578685	43627701	pseudogene	No	Yes (+)	No
18	ENSG00000263503	*RP11-707O23.5*	17	43678235	43679706	pseudogene	No	Yes (-)	No
19	ENSG00000204650	*CRHR1-IT1*	17	43697694	43725582	pseudogene	No	Yes (-)	No
20	ENSG00000185294	*SPPL2C*	17	43922256	43924438	protein_coding	No	Yes (-)	No
21	ENSG00000262500	*RP11-259G18.2*	17	44320972	44322410	pseudogene	No	Yes (-)	No
22	ENSG00000261575	*RP11-259G18.1*	17	44344403	44346060	pseudogene	No	Yes (-)	No
23	ENSG00000142273	*CBLC*	19	45281126	45303891	protein_coding	Yes	No	No
24	ENSG00000130204	*TOMM40*	19	45393826	45406946	protein_coding	Yes	No	No
25	ENSG00000130203	*APOE*	19	45409011	45412650	protein_coding	Yes	No	No
26	ENSG00000130208	*APOC1*	19	45417504	45422606	protein_coding	Yes	No	No
27	ENSG00000214855	*APOC1P1*	19	45430061	45434643	pseudogene	Yes	No	Yes
28	ENSG00000104859	*CLASRP*	19	45542298	45574214	protein_coding	No	No	Yes

Supplementary Table 5. Descriptive statistics of demographic and electronic health record features, by data sources (All of Us vs. UCLA ATLAS, stratified by the genetic inferred group)

	Hispanic Latino Americans			African Americans		
**Characteristic**	**All of Us**	**UCLA ATLAS**	**p-value**	**All of Us**	**UCLA ATLAS**	**p-value**
N	458	610	-	645	440	-
Age	75.0 (72.4, 79.0)	75.8 (72.5, 80.5)	0.1	70 (67, 75)	75.8 (72.5, 80.5)	<0.001*
Sex (Female)	288 (63%)	372 (61%)	0.5	437 (68%)	264 (60%)	0.01*
Span of records (in yrs)	6.5 (5.8, 7.8)	9.1 (6.8, 10.2)	<0.001*	6.5 (5.8, 6.8)	9.77 (7.27, 11.02)	<0.001*
Encounters per year	9 (5, 14)	14 (8, 22)	<0.001*	10 (5, 16)	13 (8, 22)	<0.001*
Number of encounters	59 (31, 105)	117 (64, 198)	<0.001*	60 (30, 108)	129 (71, 209)	<0.001*
Number of unique diagnosis	46 (28, 68)	71 (45, 109)	<0.001*	45 (28, 67)	71 (45, 103)	<0.001*
Dementia (Yes)	68 (15%)	126 (21%)	0.02*	129 (20%)	84 (19%)	0.7
APOE-ε4 count			<0.001*			<0.001*
0	425 (93%)	491 (80%)		517 (80%)	284 (65%)	
1	32 (7.0%)	117 (19%)		121 (19%)	137 (31%)	
2	1 (0.2%)	2 (0.3%)		7 (1.1%)	19 (4.3%)	

Notes: Continuous variables were reported as median (IQR), and categorical variables were reported as n (%). P-values were calcualted based on Wilcoxon rank sum test or Pearson's Chi-squared test as appropriate. * Statistical siginificant at level 0.05.

Supplementary Table 6. Descriptive statistics of demographic and electronic health record features by case/control groups, All of Us sample, stratified by genetic ancestry

	**Hispanic Latino Americans (N = 458)**			**African Americans (N = 645)**		
	**Cases**	**Controls**	**P value**	**Cases**	**Controls**	**P value**
N	68	390	-	129	516	-
Age	76.1 (70.1, 80.6)	74.9 (72.8, 78.9)	0.5	70.6 (65.8, 77.1)	70.0 (67.7, 74.8)	0.5
Sex (Female)	42 (62%)	246 (63%)	0.8	81 (63%)	356 (69%)	0.20
Span of records (in yrs)	4.5 (2.8, 6.3)	6.6 (6.0, 8.1)	<0.001*	4.0 (2.1, 6.1)	6.6 (6.2, 7.2)	<0.001*
Encounters per year	10 (4, 19)	8 (5, 14)	0.2	15 (7, 23)	9 (5, 15)	<0.001*
Number of encounters	36 (15, 78)	63 (34, 108)	<0.001*	49 (20, 89)	65 (34, 110)	<0.001*
Number of unique diagnosis	52 (27, 69)	46 (28, 68)	0.9	53 (32, 74)	43 (27, 65)	0.01*

Notes: Continuous variables were reported as median (IQR), and categorical variables were reported as n (%). P-values were calcualted based on Wilcoxon rank sum test or Pearson's Chi-squared test as appropriate. * Statistical siginificant at level 0.05.

Supplementary Table 7. Pearson's correlation of polygenic risk scores that built with genome-wide-significant or gene-annotated SNPs, UCLA ATLAS sample, stratified by genetic inferred

	**Pearson's Correlation**	
**Polygenic risk score GWAS**	**Hispanic Latino American**	**African American**
Alzheimer's Disease (European ancestry)	0.885	0.837
Alzheimer's Disease (African ancestry)	0.953	0.963
Alzheimer's Disease (multi-ancestry)	0.953	0.919
Lewy Body Dementia	0.714	0.698
Parkinson's Disease	0.561	0.29
Progressive Supranuclear Palsy	0.813	0.657
Stroke	0.737	0.669

Abbreviations: GWAS, genome-wide association study, SNP, Single-Nucleotide Polymorphism.

Supplementary Table 8. GWAS information and summary results of FUMA by phenotype

GWAS information				# SNPs (overlapped with UCLA and AOU data[b])		
**Phenotype**	**Summary statistics**	**1000G reference[a]**	**N case/control**	**Candidate**	**Independent P-significant**	**Independent gene-annotated**
Alzheimer's Disease	Kunkle et al. (2019)	EUR	21,982/41,944	1744	76	75
	Kunkle et al. (2021)	AFR	2,784/5,222	80	11	11
	Jun et al. (2017)	ALL	15,579/17,690	760	54	54
Parkinson's Disease	Nalls et al. (2019)	EUR	Meta-analysis	2640	27	27
Progressive Supranuclear Palsy	Chen et al. (2018)	EUR	1,646/10,662	732	21	21
Lewy Body Dementia	Chia et al. (2021)	EUR	2,981/4,391	356	9	9
Stroke	Malik et al. (2018)	ALL	67,162/454,450	903	21	20

Abbreviations: AFR, African American; EUR, European; GWAS, genome-wide association study; SNP, Single-Nucleotide Polymorphism.

Notes: [a] Reference panel population used for regional linkage disequilibrium patterns in identifying independent SNPs and lead P-significant SNPs. [b] Numbers of features used in PRSs building, feature selection for modeling, and phenotype prediction in the following steps.

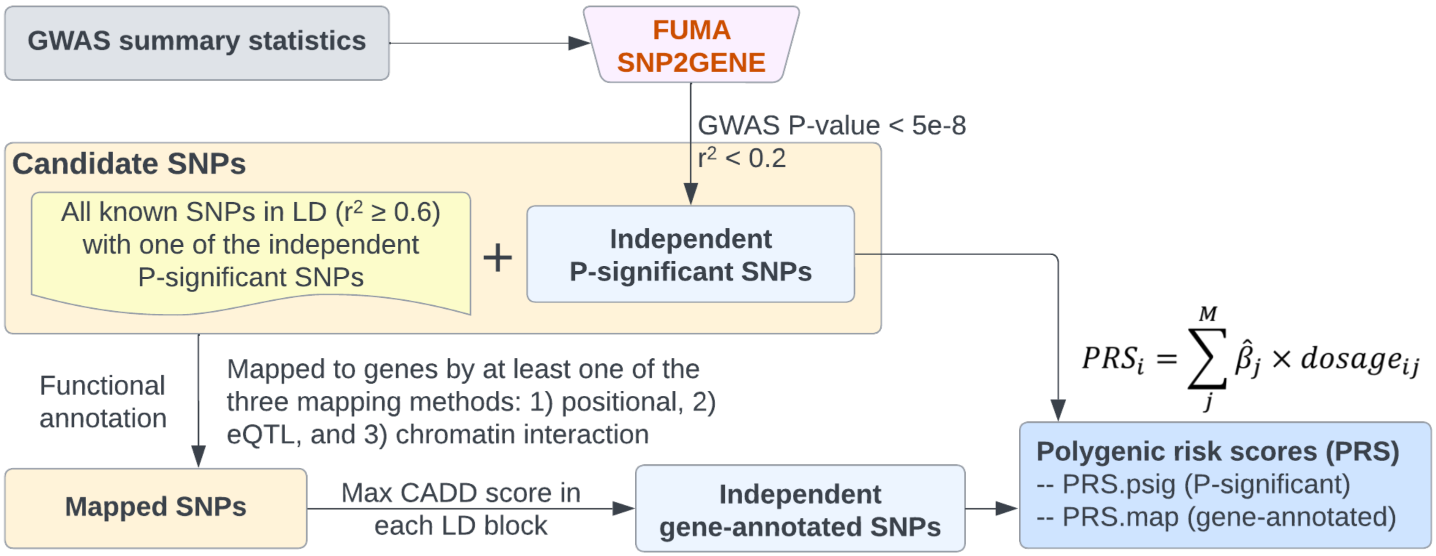

Supplementary Figure 2. Workflow of candidate SNP selection. Functional annotation and prioritization of SNPs using FUMA. Two distinct sets of SNPs (independent genome-wide-significant SNPs and independent gene-annotated SNPs) were identified by FUMA and subsequently used in our polygenic risk scores building and modeling steps. *Abbreviations: CADD, Combined Annotation Dependent Depletion; GWAS, genome-wide association study; LD, linkage disequilibrium; PRS, polygenic risk score; SNP, Single-Nucleotide Polymorphism.*

Supplementary Table 9. ICD-10 codes used for dementia phenotype definiiton

ICD-10 code	Description
**F01**	**Vascular dementia**
F01.5	Multi-infarct dementia
F01.50	Multi-infarct dementia, unspecified
F01.51	Multi-infarct dementia with delirium
F01.511	Multi-infarct dementia with delirium in Alzheimer's disease
F01.518	Multi-infarct dementia with delirium, not elsewhere classified
**F02.8***	**Dementia in other diseases classified elsewhere**
F02.80	Dementia in other diseases classified elsewhere, without behavioral disturbance
F02.81	Dementia in other diseases classified elsewhere, with behavioral disturbance
F02.811	Dementia in other diseases classified elsewhere, with behavioral disturbance in Alzheimer's disease
F02.818	Dementia in other diseases classified elsewhere, with behavioral disturbance, not elsewhere classified
**F03**	**Unspecified dementia**
F03.9	Unspecified dementia without behavioral disturbance
F03.90	Unspecified dementia with delirium
F03.91	Unspecified dementia with delirium in Alzheimer's disease
F03.911	Unspecified dementia with delirium in Alzheimer's disease, with behavioral disturbance
F03.918	Unspecified dementia with delirium in Alzheimer's disease, not elsewhere classified
**G30**	**Alzheimer's disease**
G30.0	Alzheimer's disease with early onset
G30.1	Alzheimer's disease with late onset
G30.8	Other Alzheimer's disease
G30.9	Alzheimer's disease, unspecified
**G31**	**Other degenerative diseases of nervous system, not elsewhere classified**
G31.0	Pick's disease
G31.01	Pick's disease with early onset
G31.09	Pick's disease with late onset
G31.1	Frontotemporal dementia
G31.83	Dementia with Lewy bodies
G31.85	Corticobasal degeneration
G23.1	Parkinson's disease with dementia

AA	African American
AD	Alzheimer's disease
APOE	Apolipoprotein E
AUPRC	Area Under the Precision-Recall Curve
AUROC	area under the receiver operating characteristic
CADD	Combined Annotation-Dependent Depletion
CI	confidence intervals
EA	European American
EAA	East Asian American
EHR	Electronic Health Records
FTD	Frontotemporal dementia
FUMA	Functional Mapping and Annotation of Genome-Wide Association Studies
GIA	Genetic Inferred Ancestry
GO	Gene Ontology
GWAS	Genome-Wide Association Studies
HLA	Hispanic Latino American
LBD	Lewy body dementia
LD	Linkage disequilibrium
MCC	Matthews Correlation Coefficient
PC	principal components
PDD	Parkinson’s disease dementia
PRS	Polygenic risk scores
SAA	South Asian American
SNP	Single-Nucleotide Polymorphisms
