# Supplementary material for: Improving genetic risk modeling of dementia from real-world data in underrepresented populations
Source: Commun Biol. 2024 Aug 25;7:1049. doi: 10.1038/s42003-024-06742-0 (PMC11345412; doi:10.1038/s42003-024-06742-0)
Supplement: Supplementary file 3 — Reporting Summary [file 42003_2024_6742_MOESM3_ESM.pdf]

nature portfolio

Corresponding author(s): Timothy Chang

Last updated by author(s): Jul 16, 2024

Reporting Summary

Nature Portfolio wishes to improve the reproducibility of the work that we publish. This form provides structure for consistency and transparency in reporting. For further information on Nature Portfolio policies, see our Editorial Policies and the Editorial Policy Checklist.

Statistics

For all statistical analyses, confirm that the following items are present in the figure legend, table legend, main text, or Methods section.

n/a	Confirmed	
☐	☒	The exact sample size (*n*) for each experimental group/condition, given as a discrete number and unit of measurement
☐	☒	A statement on whether measurements were taken from distinct samples or whether the same sample was measured repeatedly
☐	☒	The statistical test(s) used AND whether they are one- or two-sided<br>*Only common tests should be described solely by name; describe more complex techniques in the Methods section.*
☐	☒	A description of all covariates tested
☐	☒	A description of any assumptions or corrections, such as tests of normality and adjustment for multiple comparisons
☐	☒	A full description of the statistical parameters including central tendency (e.g. means) or other basic estimates (e.g. regression coefficient) AND variation (e.g. standard deviation) or associated estimates of uncertainty (e.g. confidence intervals)
☐	☒	For null hypothesis testing, the test statistic (e.g. *F*, *t*, *r*) with confidence intervals, effect sizes, degrees of freedom and *P* value noted<br>*Give P values as exact values whenever suitable.*
☒	☐	For Bayesian analysis, information on the choice of priors and Markov chain Monte Carlo settings
☒	☐	For hierarchical and complex designs, identification of the appropriate level for tests and full reporting of outcomes
☐	☒	Estimates of effect sizes (e.g. Cohen's *d*, Pearson's *r*), indicating how they were calculated

Our web collection on statistics for biologists contains articles on many of the points above.

Software and code

Policy information about availability of computer code

Data collection	We used Electronic Health Records collected by the UCLA Health team.
Data analysis	All analyses were conducted using the R software. Code is available on GitHub: https://github.com/TSChang-Lab/Dementia-prediction. Requests for additional information can be directed to the Lead Contact: Timothy S Chang (timothychang@mednet.ucla.edu).

For manuscripts utilizing custom algorithms or software that are central to the research but not yet described in published literature, software must be made available to editors and reviewers. We strongly encourage code deposition in a community repository (e.g. GitHub). See the Nature Portfolio guidelines for submitting code & software for further information.

Data

Policy information about availability of data

All manuscripts must include a data availability statement. This statement should provide the following information, where applicable:

- Accession codes, unique identifiers, or web links for publicly available datasets
- A description of any restrictions on data availability
- For clinical datasets or third party data, please ensure that the statement adheres to our policy

The Genome-Wide Association Study summary statistics data analyzed in this study are publicly available. Individual electronic health record data are not publicly available due to patient confidentiality and security concerns. Collaboration with the study authors who have been approved by UCLA Health for Institutional

Review Board-qualified studies are possible and encouraged. Code is available on GitHub: https://github.com/TSChang-Lab/Dementia-prediction. Requests for additional information can be directed to the Lead Contact: Timothy S Chang (timothychang@mednet.ucla.edu).

Human research participants

Policy information about studies involving human research participants and Sex and Gender in Research.

Reporting on sex and gender	Self-reported gender was used in the analysis.
Population characteristics	Self-reported age, gender were used in the analysis. Genetic Inferred Ancestry was inferred using the reference panel from the 1000 Genomes Project and principal component analysis.
Recruitment	Participants were recruited from the UCLA Health System. Patient consent was obtained before collecting bio-sample.
Ethics oversight	As the genetic data and EHRs were de-identified, the study was exempt from human subject research regulations (UCLA IRB# 21-000435).

Note that full information on the approval of the study protocol must also be provided in the manuscript.

Field-specific reporting

Please select the one below that is the best fit for your research. If you are not sure, read the appropriate sections before making your selection.

☒ Life sciences ☐ Behavioural & social sciences ☐ Ecological, evolutionary & environmental sciences

For a reference copy of the document with all sections, see nature.com/documents/nr-reporting-summary-flat.pdf

Life sciences study design

All studies must disclose on these points even when the disclosure is negative.

Sample size	No sample size was calculated. We used all eligible data that met the inclusion criteria from the UCLA Health System.
Data exclusions	We excluded patients based on their age and exclusion phenotypes to ensure true cases and controls.
Replication	We replicated our primary findings from the UCLA sample in a comparable sample from the All of Us database.
Randomization	Samples were randomized to folds to perform cross-validation in the modeling steps.
Blinding	We collected data directly from the Electronic Health Records and linked biobank.

Reporting for specific materials, systems and methods

We require information from authors about some types of materials, experimental systems and methods used in many studies. Here, indicate whether each material, system or method listed is relevant to your study. If you are not sure if a list item applies to your research, read the appropriate section before selecting a response.

Materials & experimental systems

n/a	Involved in the study	
☒	☐	Antibodies
☒	☐	Eukaryotic cell lines
☒	☐	Palaeontology and archaeology
☒	☐	Animals and other organisms
☒	☐	Clinical data
☒	☐	Dual use research of concern

Methods

n/a	Involved in the study	
☒	☐	ChIP-seq
☒	☐	Flow cytometry
☒	☐	MRI-based neuroimaging
